# Effect of Dapagliflozin on Patients with Rheumatic Heart Disease Mitral Stenosis

**DOI:** 10.3390/jcm12185898

**Published:** 2023-09-11

**Authors:** An Aldia Asrial, Reviono Reviono, Soetrisno Soetrisno, Budi Yuli Setianto, Vitri Widyaningsih, Ida Nurwati, Brian Wasita, Anggit Pudjiastuti

**Affiliations:** 1 Doctoral Program of Medical Sciences Department, Faculty of Medicine, Universitas Sebelas Maret, Surakarta 57126, Indonesia; 2 Department of Cardiology and Vascular Medicine, Faculty of Medicine, Universitas Sebelas Maret—Universitas Sebelas Maret Hospital, Surakarta 57126, Indonesia; 3 Department of Pulmonology and Respiratory Medicine, Faculty of Medicine, Universitas Sebelas Maret—Universitas Sebelas Maret Hospital, Surakarta 57126, Indonesia; 4 Department of Obstetrics and Gynecology, Faculty of Medicine, Universitas Sebelas Maret—Universitas Sebelas Maret Hospital, Surakarta 57126, Indonesia; 5 Department of Cardiology and Vascular Medicine, Faculty of Medicine, Universitas Gadjah Mada—Dr. Sardjito General Hospital, Yogyakarta 55281, Indonesia; 6 Department of Public Health, Faculty of Medicine, Universitas Sebelas Maret, Surakarta 57126, Indonesia; 7 Department of Biomedical Sciences, Faculty of Medicine, Universitas Sebelas Maret, Surakarta 57126, Indonesia; 8 Department of Pathology, Faculty of Medicine, Universitas Sebelas Maret, Surakarta 57126, Indonesia; 9 Department of Cardiology and Vascular Medicine, Permata Bunda Hospital, Purwodadi 58114, Indonesia

**Keywords:** dapagliflozin, rheumatic heart disease, mitral stenosis, mitral valve MPG, net atrioventricular compliance, NT-pro BNP

## Abstract

(1) Background: Mitral stenosis is the most common rheumatic heart disease (RHD). Inflammation and fibrosis are the primary pathophysiology, resulting in left atrial stress and dysfunction. Dapagliflozin is a new heart failure treatment with anti-inflammation and anti-fibrosis effects from previous studies. However, the specific role of dapagliflozin in RHD mitral stenosis is unknown. This study aims to investigate (i) the effect of dapagliflozin on biomarkers of fibrosis, NT-pro BNP levels and left atrial function; (ii) the relationship between the changes in fibrosis biomarkers with left atrial function and NT-pro BNP levels. (2) Methods: An open-label randomized study was conducted on 33 RHD mitral stenosis patients divided into a dapagliflozin group which received 10 mg dapagliflozin and standard therapy, and a control group which only received standard therapy. All patients were examined for levels of PICP, MMP-1/TIMP-1 ratio, TGF-β1, NT-proBNP, mitral valve mean pressure gradient (MPG), and net atrioventricular compliance (Cn) pre- and post-intervention. (3) Results: This study found a significant increase in PICP and TGF-β1 and a reduction in the MMP-1/TIMP-1 ratio in the dapagliflozin group and the control group (*p* < 0.05). In the dapagliflozin group, the levels of NT-pro BNP decreased significantly (*p* = 0.000), with a delta of decreased NT-pro BNP levels also significantly greater in the dapagliflozin group compared to the control (*p* = 0.034). There was a significant increase in Cn values in the dapagliflozin group (*p* = 0.017), whereas there was a decrease in the control group (*p* = 0.379). Delta of changes in Cn values between the dapagliflozin and control groups also showed a significant value (*p* = 0.049). The decreased MPG values of the mitral valve were found in both the dapagliflozin and control groups, with the decrease in MPG significantly greater in the dapagliflozin group (*p* = 0.031). There was no significant correlation between changes in the value of fibrosis biomarkers with Cn and NT-pro BNP (*p* > 0.05). (4) Conclusions: This study implies that the addition of dapagliflozin to standard therapy for RHD mitral stenosis patients provides benefits, as evidenced by an increase in net atrioventricular compliance and decreases in the MPG value of the mitral valve and NT-pro BNP levels (*p* < 0.05). This improvement was not directly related to changes in fibrosis biomarkers, as these biomarkers showed ongoing fibrosis even with dapagliflozin administration.

## 1. Introduction

Rheumatic heart disease (RHD) remains a significant health problem that causes mortality and morbidity, mainly in developing countries [1]. Cardiac inflammation and fibrosis of valves and myocardium are the primary manifestations. The disease begins with acute rheumatic fever and molecular mimicry between streptococcal group-A antigen and host tissue, causing activation of immune cells and leading to fibrosis and dysfunction of the valves [2,3]. Several serum biomarkers have been studied and shown to be associated with cardiac fibrosis in RHD. These fibrosis biomarkers include transforming growth factor-β1 (TGF-β1)—a marker of collagen synthesis and extracellular matrix remodeling [4,5]; circulating carboxy-terminal propeptide of type I procollagen (PICP)—a marker of type I collagen synthesis; and the ratio between matrix metalloproteinase I (MMP-1) and tissue matrix metalloproteinase inhibitors 1 (TIMP-1), which describes the balance of collagen degradation processes and inhibition [6,7]. The shift from degradation to synthesis of the extracellular matrix will determine the increase or decrease of collagen and the degree of fibrosis that occurs [8].

Fibrosis of the valve will cause valve dysfunction; one of the most common abnormalities is mitral stenosis [9,10]. In mitral stenosis, there is a disturbance in the opening of the mitral valve, which increases left atrial (LA) and pulmonary pressure and causes complaints of heart failure. In the long term, this pressure will also cause fibrosis in the left atrium. In addition to pressure factors, chronic inflammation of RHD is a stress responsible for left atrial fibrosis [11,12]. This fibrosis then causes interference in the left atrium, which can be measured using the parameter of net atrioventricular compliance (Cn). This parameter has been associated with prognosis after intervention, given its relation to pulmonary hypertension, activity intolerance and progression of mitral stenosis in medical treatments [13]. Stress on the myocardium, especially in the left atrial, also increases the level of NT-pro BNP. This biomarker has been extensively studied in heart failure patients and associated with parameters of left atrial dimension and pressure, the mitral valve area, and patient functional class in RHD mitral stenosis [14,15].

Dapagliflozin is an SGLT2 inhibitor-class drug currently used across a broad spectrum of heart failure cases [16]. Various mechanisms of these drug benefits continue to be studied, including in inflammation and fibrosis pathways [17]. The role of the SGLT2 pathway has been proven in cardiac fibrosis mainly through the collagen type I and III expression pathways found in both in vivo and in vitro studies [18,19,20]. Research on animal models of mitral regurgitation found that dapagliflozin improves left ventricular cardiac fibrosis [21]. In left atrial clinical studies, dapagliflozin was found to improve left atrial function and maximal volume and reduce the risk of atrial fibrillation/atrial flutter, which is known to be associated with atrial fibrosis [22,23,24,25].

Currently, no treatment for rheumatic heart disease mitral stenosis targets the primary pathogenesis—fibrosis. Previous studies have tried several drugs to inhibit fibrosis. However, the results are inconsistent, and these drugs have not yet become standard therapy [26,27,28]. Therefore, new approaches and treatments are needed to prevent RHD progression, and perhaps to improve LA function. The role and benefits of dapagliflozin in RHD mitral stenosis patients are not known, specifically in fibrosis pathways and left atrial function. This study aims to investigate (i) the effect of dapagliflozin on biomarkers of fibrosis, left atrial function and NT-pro BNP levels and (ii) the relationship between the changes in fibrosis biomarkers with left atrial function and NT-pro BNP levels.

## 2. Materials and Methods

### 2.1. Study Design

This study is a clinical experimental study with an open-label design, randomized, controlled trial, pre-test, and post-test design. The protocol was approved by the Faculty of Medicine Universitas Sebelas Maret Research Ethics Committee (No.128/UN27.06.11/KEP/EC.2022). The study was registered in ClinicalTrials.gov (NCT05618223). The sample was randomly divided into two groups (random assignment), namely, the dapagliflozin group (dapagliflozin and standard treatment) and the control group (standard treatment only). Subjects in the dapagliflozin group received standard medical treatment plus dapagliflozin, 10 mg/day for 4 weeks, while subjects in the control group received standard medical treatment only.

### 2.2. Subject

The study was conducted at Panti Rahayu Hospital and Permata Bunda Hospital in Purwodadi, Indonesia. The subjects included were outpatients at the Cardiology polyclinic for a primary diagnosis of mitral stenosis RHD. This diagnosis was screened with the following inclusion criteria: planimetry mitral valve area ≤ 1.5 cm^2^ in echocardiography with morphology supporting RHD (calcification and fusion of leaflets and commissures and with restrictive valve mobility) [29,30,31]; and New York Heart Association functional class 2–3. 

Exclusion criteria included significant (moderate to severe) mitral and aortic valve disease besides mitral stenosis; patients who were pregnant or breastfeeding; patients who were hemodynamically unstable or experiencing severe acute decompensation characterized by signs of congestion in the form of crackles of more than one-third of the lung fields, ascites, and/or signs and symptoms of cardiogenic/hypovolemic shock; patients after mitral valve replacement surgery or after percutaneous balloon mitral valvuloplasty; patients known to be allergic to SGLT2 inhibitors; type 1 diabetes mellitus; patients currently undergoing treatment with SGLT2 inhibitors or having received SGLT2 inhibitor therapy in the last 4 weeks; patients with a history of more than one episode of severe hypoglycemia (GDS < 60 mg/dl) on insulin or sulfonylurea treatment; patients with chronic kidney disease stage IV (estimated glomerular filtration rate (eGFR) = 15–29 mL/min/1.73 m^2^) and/or stage V (eGFR < 15 mL/min/1.73 m^2^) and/or who are undergoing dialysis (hemodialysis); and patients with severe lung disease.

### 2.3. Measurement of Biomarker and LA Function

The venous blood sample of each subject was collected into a separate serum tube pre- and post-intervention. The research protocol of TGF-β1, PICP, MMP-1, TIMP-1 and NT-pro BNP levels used the ELISA method and was conducted per the manufacturer’s instructions. The ELISA kits used were: Elikine^TM^ Human TGF-β1 ELISA Kit (KET6030) (Abbkine, Atlanta, GA, USA), ABclonal Human PICP chain ELISA Kit (RK09063) (Abclonal, Woburn, MA, USA), ABclonal Human Total MMP-1 ELISA Kit (RK00340), Elikine^TM^ Human TIMP ELISA Kit (KET6031), ABclonal Human NT-pro BNP ELISA Kit (RK09256). The fibrosis biomarkers and NT-pro BNP examination were carried out in the biomedical laboratory of Sebelas Maret University. 

Each well contained 100 μL of standard and human serum incubated for 2 h at 37 °C. After washing three times, 100 μL working biotin conjugate antibody was added to the well and set for 1 h at 37 °C. Then, each well received 100 μL working streptavidin-HRP, 90 μL substrate solution and 50 μL stop solution. The final step was to detect optical density within 5–30 min at a wavelength under 450 nm. All standard equipment, including well, microplate reader, multi-channel pipette, incubator, precision pipettes and water, was provided by the biomedical laboratory of Sebelas Maret University.

Standard echocardiography examination to evaluate RHD mitral stenosis was performed pre- and post-intervention.

Left atrial function assessment was performed using an echocardiography General Electric Echocardiography Vivid T8 machine. Each patient was examined by standard echocardiography to evaluate mitral stenosis, and left atrial function was evaluated by measuring the net atrioventricular compliance value and mitral-valve mean pressure gradient. Net atrioventricular compliance was calculated using the following formula:

Cn (mL/mmHg) =−1270 × mitral valve area (MVA) (cm^2^)/E-wave downslope (cm/s^2^).

The value of the mitral valve area was obtained from an echocardiographic examination, using the planimetry method on a parasternal short-axis view at the level of the mitral valve [32,33]. The E-wave downslope value was obtained from an echocardiographic examination using a pulsed wave Doppler at the apical 4-chamber view. In patients with atrial fibrillation, a pulsed-wave Doppler examination was performed 5 times (5 cardiac cycles), and the E-wave downslope value was the average value of the 5 cardiac cycles [34]. After obtaining the mitral valve area and e-wave downslope values, Cn was calculated manually. Mitral valve MPG examination was performed using echocardiography by placing a marker on the tip of the mitral valve in 4-chamber view. Volume sampling was carried out using a continuous wave Doppler to obtain the MPG value of the mitral valve [31].

### 2.4. Statistical Analysis

The normality test was conducted on each data element to see the distribution. The normality test used the Shapiro–Wilk test, with *p* > 0.05 indicating normal data distribution. For normally distributed data, the test for different means of pre- and post-intervention values in one group was carried out by a paired *t*-test. An unpaired *t*-test was carried out to test differences in means between groups. For data that were not normally distributed, a different test of the means of pre- and post-intervention values in one group was performed by the Wilcoxon signed rank test, while the Mann–Whitney test was carried out to test different means between groups. The correlation between two continuous variables was measured by Pearson’s correlation test. The *p*-value is considered significant if it is less than 0.05. Data analysis was performed using IBM^®^ SPSS^®^ statistics version 25.

## 3. Results

Thirty-three patients were enrolled (17 patients in the dapagliflozin group and 16 in the control group), with similar baseline characteristics (Table 1).

### 3.1. Effect of Dapagliflozin on Biomarker Fibrosis Levels in RHD Mitral Stenosis 

This study found a significant increase in PICP and TGF-β1 values post-intervention in the dapagliflozin and control groups (*p* = 0.000). Meanwhile, in the MMP-1/TIMP-1 ratio, there was a significant decrease in the dapagliflozin group (*p* = 0.005) and the control group (*p* = 0.002). The delta changes of PICP, TGF-β1, and the MMP-1/TIMP-1 ratio were not significantly different between the dapagliflozin and control groups. These results confirmed that the fibrosis process was still ongoing, and that administration of dapagliflozin had not been shown to inhibit the increase in PICP and TGF-β1 and decrease the MMP-1/TIMP-1 ratio (Table 2).

### 3.2. Effect of Dapagliflozin on NT-pro BNP Levels in RHD Mitral Stenosis

**Figure 1:**
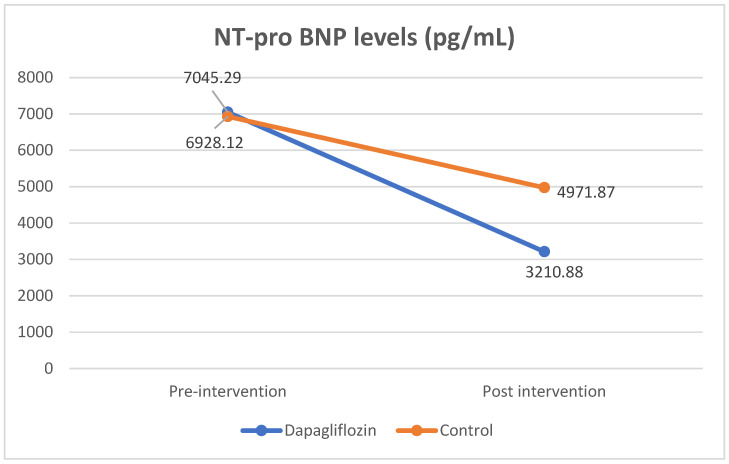
Comparison of NT-pro BNP levels pre- and post-intervention.

Results indicating positive effects of dapagliflozin were obtained as to NT-pro BNP levels. There was a significant reduction in NT-pro BNP levels in both the dapagliflozin and control groups. In the dapagliflozin group, the levels of NT-pro BNP decreased significantly, from 7045.29 ± 3182.26 pg/mL to 3210.88 ± 1019.46 pg/mL (*p* = 0.000). In the control group, the level of NT-pro BNP also decreased significantly, from 6928.12 ± 3690.44 pg/mL to 4971.87 ± 3634.65 mg/dL (*p* = 0.002). A significant difference in NT-pro BNP levels was also found in the delta of decreased NT-pro BNP levels in the treatment group compared to the control (3832.42 ± 2857.52 vs 1956.25 ± 1755.42; *p* = 0.034). From the results of this analysis, it was found that dapagliflozin and standard medication significantly reduced levels of NT-pro BNP. Even so, dapagliflozin administration reduced NT-pro BNP levels more than the reduction in the control group (Table 3 and Figure 1).

### 3.3. Effect of Dapagliflozin on Cn and Mitral Valve Mean Pressure Gradient in RHD Mitral Stenosis

We found no significant differences in echocardiographic parameters in the dapagliflozin and control groups pre- and post-intervention. When analyzing the differences in parameters within each group, we found no significant differences except for the net atrioventricular compliance and mitral valve mean pressure gradient parameters. There was a reduction in RV diameter and LAVI, but it was not statistically significant (Table 4). 

**Figure 2:**
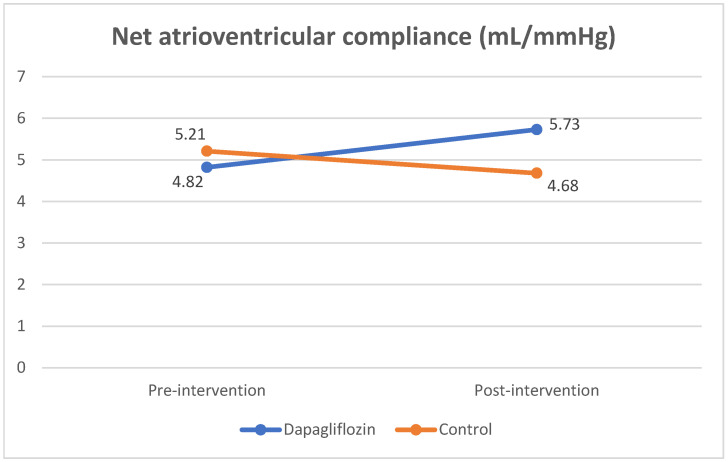
Comparison of net atrioventricular compliance (Cn) value pre- and post-intervention.

Although the fibrosis biomarkers did not show inhibition of the fibrotic process, we found an increase in left atrial function, as measured by the Cn value. There was a significant increase in Cn values in the post-intervention dapagliflozin group ((4.82 ± 1.71 to 5.73 ± 2.19 mL/mmHg; *p* = 0.017). In the control group, there was a decrease in post-intervention Cn values (5.21 ± 1.99 to 4.68 ± 1.73 mL/mmHg; *p* = 0.379). There was a significant difference in the delta changes in Cn values between the dapagliflozin and control groups (*p* = 0.049) (Table 5 and Figure 2).

The mitral valve mean pressure gradient parameter also showed a significant improvement in the dapagliflozin group, as compared to the control group. A decrease in the MPG value of the mitral valve was found in both the dapagliflozin group and the control group (3.34 ± 3.11 and 0.46 ± 4.15 mmHg), but the decrease in MPG was significantly greater in the dapagliflozin group (*p* = 0.031 (Table 6).

**Figure 3:**
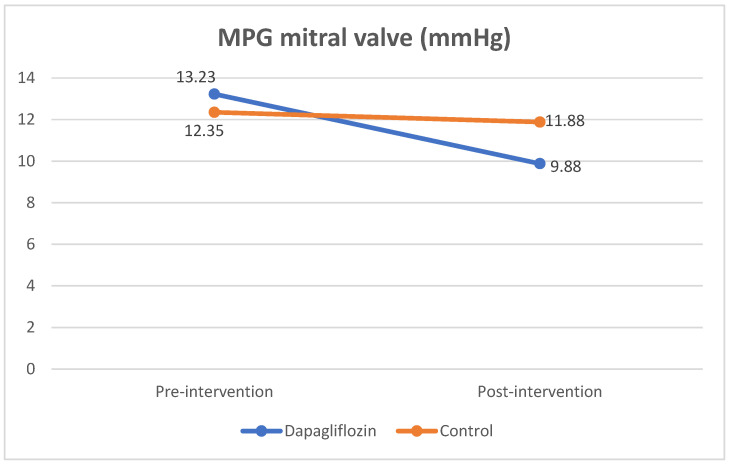
Comparison of MPG mitral-valve value pre- and post-intervention.

In subsequent analyses, we found a significant association between change in Cn values and mitral valve MPG (r = −0.463; *p* = 0.007). Better Cn is associated with lower mitral-valve MPG values (Figure 3). 

### 3.4. Relationship of Changes in Fibrosis Biomarkers with Cn and NT-pro BNP Levels

In this study, we did not find a significant correlation between changes in the values of the biomarkers PICP, MMP-1/TIMP-1 ratio, or TGF-β1 with Cn (PICP with Cn (r = −0.297; *p* = 0.093); MMP-1/TIMP-1 with Cn (r = −0.056; *p* = 0.756); TGF-β1 with Cn (r = 0.057; *p* = 0.751)).

There was also no correlation between changes in fibrosis biomarker values and NT-pro BNP levels (PICP with NT-pro BNP (r = −0.240; *p* = 0.354), MMP-1/TIMP-1 ratio with NT-pro BNP (r = 0.330; *p* = 0.196); TGF-β1 with NT-pro BNP (r = −0.302; *p* = 0.238)).

## 4. Discussion

The anti-fibrosis effects of dapagliflozin have been demonstrated in several studies. Ye et al. (2017) found that dapagliflozin attenuated the activation of the inflammasome, fibrosis, and deterioration of LVEF in BTBR mice model cardiomyopathy. Dapagliflozin significantly attenuated the elevated mRNA levels of NALP3, ASC, IL-1β, IL-6, caspase-1, and TNFα in the BTBR mice model. Then, dapagliflozin also significantly attenuated the increase in type I and type III collagen mRNA levels and reduced the percentage of fibrosis on Masson’s trichrome staining [35].

Activation of the TGFβ1/Smad signaling pathway is one of the main pathways of cardiac fibrosis. Research by Zhang et al. (2021) found that administration of dapagliflozin inhibited cardiac fibroblast (CF) collagen production induced by angiotensin II in vitro by regulating TGF-β1/Smads signaling. Dapagliflozin pretreatment inhibited left ventricular dysfunction, left ventricular hypertrophy, fibrosis, and collagen synthesis induced by angiotensin II [20]. Meanwhile, in a study by Chen et al. (2022), inhibition of this pathway by dapagliflozin reduced the expression of MMP-2, MMP-9 and TIMP-1 (*p* < 0.05), thereby improving fibrosis in normoglycemic heart failure rabbit models [36].

However, the anti-fibrotic effects of dapagliflozin were not proved in this study. The treatment group with dapagliflozin did not show significant inhibition in increasing PICP and TGF-β1 levels and decreasing the MMP-1/TIMP-1 ratio. There are several possibilities to explain why this inhibitory effect was not proved in this study. Dapagliflozin has improved fibrosis through the TGFβ1/Smad signaling pathway, the NLRP3/ASC inflammasome, or the mitogen-activated protein kinase (MAPK) signaling pathway [18,19,20,35,36]. Nevertheless, the fibrosis signaling pathway for rheumatic heart disease is still being studied, and other pathways may play a role beyond those already known. A previous review by Xian and Zheng (2021) has identified three intervention targets that can be used for the treatment of RHD: interventions in IFN-γ and TNF-α--mediated ECM remodeling, suppression of α-SMA expression in TGF-β1-induced fibroblasts via the AKT/S6K pathway and disruption of STAT3 phosphorylation to prevent cytokine release from Th17 cells and reduce induction of valve damage by RHD. However, it is not yet known how much impact intervention in that specific pathway will have on fibrosis in RHD mitral stenosis, and further study is needed [37].

This study is also based on several previous studies related to the use of dapagliflozin in human heart failure patients, in which significant clinical benefits have been seen on the 28th day [38]. Even so, specifically regarding the intervention for fibrosis by administering dapagliflozin, several animal studies were carried out over a more extended period [19,35]. The duration of dapagliflozin use and the emergence of anti-inflammatory and anti-fibrosis effects in humans are unknown. A longer intervention time may be needed to see the impact of dapagliflozin on biochemical marker parameters of fibrosis, although left atrial function has shown significant changes.

The increase in fibrosis biomarkers can be caused by the fibrosis process in the valves and myocardium atrial and ventricles. Valves are structurally distinct from the myocardium, including their response to inflammation and fibrosis in RHD. The fibrotic response in the valves is more severe and causes permanent damage. One of the hypotheses that explain this valve damage refers to the lower level of anti-inflammatory cytokine (IL-4) in the valve compared to the myocardium [39,40]. Thus far, research on dapagliflozin referencing heart disease has mainly been carried out to target the myocardium. The biochemical markers of fibrosis used in this study are circulating markers whose increase can occur by the increasing fibrosis of either the valves or the myocardium.

Sodium–glucose transporters mediate apical sodium and glucose transport across cell membranes and are also known as Na^+^/glucose co-transporters or symporters (SGLTs). SGLT2 is a member of the SLC5 gene family, a subdivision of an ancient superfamily of sodium co-transporters [41]. Until now, the expression of SGLT2 receptors in valvular areas has been unknown. SGLT2 is mainly expressed in the kidney, and is located in the first part of the proximal tubule, which allows ∼90% of glucose reabsorption from the urine. The SGLT2 receptors have not been detected in cardiomyocytes but are known to directly affect the heart [42]. 

The mechanism of action of dapagliflozin in heart failure is still a question, and the research is continuing. In addition to the inflammatory and fibrotic pathways, there are several other hypotheses, such as their effect on cardiac metabolism and myocardial bioenergetics, changes in adipokines and epicardial adipose tissue mass, as well as their impact on loading conditions mainly through the natriuresis–diuresis pathway [43]. A comparative study by Wilcox et al. (2018) [44] found that dapagliflozin has the same sodium-reducing effect and interstitial fluid volume as the loop diuretic bumetanide but without a significant change in intravascular volume. Another study by Heerspink et al. (2013) [45] found that giving dapagliflozin for 12 weeks compared to hydrochlorothiazide reduced plasma volume and increased erythrocyte mass. Interstitial volume regulation is important for patients with heart failure, including patients with RHD mitral stenosis. Compared to conventional diuretic drugs, which cause a decrease in interstitial and intravascular volume, the selective effect of dapagliflozin on interstitial volume without interference with intravascular volume will be beneficial. This selective effect does not cause reflex neurohumoral stimulation, which can exacerbate heart failure [43,44,45]. Fluid volume is an essential component of left atrial function. At the same MVA value, MPG will be directly proportional to the fluid volume and inversely proportional to the filling time of the ventricles in the diastole phase [46]. Fluid volume, left atrial volume, trans-mitral blood flow (mean pressure gradient mitral valve) and Cn are interrelated factors associated with left atrial mechanical dysfunction [47,48].

Regarding the role of dapagliflozin in improving cardiac chamber pressure, especially the left atrium, similar results were obtained in the phase II randomized clinical trial “Evaluation of the Cardiac and Metabolic Effects of Dapagliflozin in Heart Failure with Preserved Ejection Fraction” (CAMEO-DAPA). In this study, dapagliflozin was administered to heart failure patients with preserved ejection fraction. During the 24-week observation period, a significant decrease in pulmonary capillary wedge pressure (PCWP) was found, reflecting left atrial pressure in the dapagliflozin treatment group either at rest (∆ absolute difference: –3.5 mmHg; 95% CI: –6.7 to –0.4; *p* = 0.029) or during activity (∆ absolute difference: –6.1 mmHg; 95% CI: –11.2 to –1.0; *p* = 0.019). Dapagliflozin also significantly reduced right atrial and pulmonary artery pressure during activity, plasma volume and body weight [49]. Another research trial on the “Impact of Dapagliflozin on Left Ventricular Diastolic Dysfunction in Patients with Type 2 Diabetes Mellitus” (IDDIA) also showed the dapagliflozin effect on left ventricular pressure. Dapagliflozin administration in type 2 diabetes patients with standard therapy was associated with significantly improved left ventricular diastolic function and decreased estimated LV filling pressure on exercise [50]. Another mechanism that can explain the improvement of myocardial function is the ion pathway and endothelium function. Research by Cappetta et al. [51] in Dahl rats showed that dapagliflozin reduced Ca^2+^ and Na^+^ overload and prevented decreased Ca^2+^ transient amplitude. Dapagliflozin was also found to improve endothelial function, as evidenced by a decrease in markers of endothelial activation. Dapagliflozin was further shown to partially restore endothelial nitric oxide synthase, which was downregulated in diastolic dysfunction (*p* < 0.05) [51].

We also found a significant decrease in NT-pro BNP levels in the dapagliflozin group. The NT-pro BNP value describes the level of myocardial stress; in previous studies, the NT-pro BNP value has been correlated with echocardiography parameters and the patient’s functional class [15]. The study by Iltumur et al. (2005) also found that NT-pro BNP levels correlated positively with the severity of mitral stenosis and pulmonary artery pressure and negatively correlated with the mitral valve area (MVA) (*p* < 0.001) [14]. The NT-pro BNP value can also explain the relationship between hemodynamic status and patient symptoms, so this parameter can be used to monitor the progression and clinical severity of RHD mitral stenosis [52]. The role of NT-pro BNP is also influenced by the management carried out; in the study of Safi et al. (2017), a significant decrease in NT-pro BNP was found after the percutaneous mitral commissurotomy intervention in RHD mitral stenosis patients, and the decrease in value correlated with a decrease in the mean pressure gradient (MPG) [53].

Based on several previous studies, the ventricular myocardium is known to be the main source of BNP. However, other studies have uncovered the possibility of different sites synthesizing or producing BNP. Research by Khare and Dwivedi (2016) found a correlation between left atrial dysfunction examined by tissue-Doppler-derived strain/strain rate (S/Sr) and NT-pro BNP levels [54]. This hormone level can also be used to predict improvement in left atrial function after percutaneous mitral balloon valvuloplasty. In another study with lone atrial fibrillation patients, blood samples taken from the coronary sinus showed levels of NT-pro BNP higher than those of the aorta and anterior interventricular vein (AIV), where samples from the coronary sinus indicated an NT-pro BNP value in the atrium [55].

This finding was supported by a study by Sharma et al. (2011) [56] in RHD mitral stenosis patients; it was found that both BNP and atrial natriuretic peptide (ANP) were associated with disease severity, but ANP was not shown to be significantly related to exercise capacity or increased blood pressure during exercise. In contrast, increased BNP was associated with a lower left atrial area index, lower exercise capacity, and higher pulmonary artery pressure [54]. A decrease in the value of NT-pro BNP in our study could indicate a decrease in myocardial stress, specifically in the left atrial myocardium [56].

In general, these results open up new potential beneficial effects of dapagliflozin administration in patients with RHD mitral stenosis. It is hoped that improving Cn function, decreasing MPG of the mitral valve, and NT-pro BNP levels will help reduce signs and symptoms of heart failure in patients.

There are several limitations to this research. This research was conducted on a small sample size as a preliminary study. The intervention period was also short, so in the future, it could be carried out on a larger number of subjects and for a longer duration. In addition, the biomarkers of fibrosis that were examined are biomarkers circulating in the circulation, so there is still the possibility of being influenced by other factors; thus, an assessment with other methods is needed to assess fibrosis in the left atrium, for example, with the Cardiac magnetic resonance imaging (CMR). Other mechanisms related to improving left atrial function besides the fibrosis pathway also need to be investigated to find out the mechanism for the improvement of left atrial function in this study, even though no inhibition was found in the fibrosis process. Further in-depth research is needed to understand the mechanism of dapagliflozin’s beneficial effect in RHD mitral stenosis patients.

## 5. Conclusions

Administration of dapagliflozin in RHD mitral stenosis patients has been shown to improve left atrial function, as evidenced by improvements in Cn, the MPG value of the mitral valve, and NT-pro BNP levels. This improvement was not directly related to changes in fibrosis biomarkers, as these biomarkers showed ongoing fibrosis, even with dapagliflozin administration.

## Figures and Tables

**Table 1:** Group characteristics and baseline data.

Patients	Dapagliflozin Group (*n* = 17)	Control Group (*n* = 16)	*p*-Value
Demography and comorbidities			
Age, years	51.35 ± 9.88	55.94 ± 6.65	0.13
Sex			
Female, *n* (%)	14 (82.53%)	15 (93.75%)	0.601
Male, *n* (%)	3 (17.64%)	1 (6.25%)	
Body mass index (BMI), kg/m^2^	22.87 ± 3.14	21.08 ± 3.10	0.11
Atrial fibrillation (%)	17 (100%)	16 (100%)	-
Hypertension, *n* (%)	1 (5.88%)	3 (1.87%)	0.335
Type 2 diabetes, *n* (%)	2 (11.76%)	0 (0%)	0.485
Coronary artery disease, *n* (%)	0 (0%)	0 (0%)	-
Smoker, *n* (%)	1 (5.88%)	1 (6.25%)	0.965
Examination			
Systolic blood pressure, mmHg	118.53 ± 13.25	116.63 ± 17.25	0.417
Diastolic blood pressure, mmHg	76.71 ± 10.83	78.00 ± 13.79	0.766
Heart rate, bpm	72.11 ± 14.13	78.93 ± 15.50	0.196
Creatinine, mg/dL	1.03 ± 0.48	0.93 ± 0.34	0.773
eGFR, mL/ mL/min/1.73 m^2^	67.94 ± 26.37	65.37 ± 22.22	0.765
Blood glucose, mg/dL	128.94 ± 43.78	116.75 ± 22.22	0.787
Echocardiography parameters			
MVA planimetry, cm^2^	0.75 ± 0.13	0.77 ± 0.13	0.616
LA diameter, mm	56.14 ± 9.26	53.52 ± 8.36	0.402
RV diameter, mm	36.29 ± 4.51	33.46 ± 7.09	0.177
LVIDd, mm	47.34 ± 7.21	45.99 ± 5.81	0.665
LAVI, mL/m^2^	145.89 ± 75.29	139.26 ± 69.27	0.707
Cn, mLmmHg	4.82 ± 1.71	5.21 ± 1.99	0.546
Mean pressure gradient mitral, mmHg	13.23 ± 4.50	12.35 ± 4.48	0.579
Systolic pulmonary artery pressure, mmHg	64.83 ± 14.74	65.65 ± 22.79	0.902
LVEF, %	53.73 ± 10.36	57.20 ± 9.02	0.314
TAPSE, mm	18.30 ± 4.12	18.42 ± 7.53	0.954
Pulmonary hypertension probability (intermediate to high), %	15 (88.23%)	12 (75%)	0.398
Pharmacological treatment			
ACE-I/ ARB	0 (0%)	0 (0%)	-
Beta blockers	12 (70.58%)	8 (50%)	0.394
Furosemide	13 (76.47%)	12 (75%)	1
Spironolactone	17 (100%)	16 (100%)	-
Antiplatelet	0 (0%)	0 (0%)	-
Warfarin	17 (100%)	16 (100%)	-
Digoxin	5 (29.41%)	8 (50%)	0.394
Complication			
Hypoglycemia	1 (5.88%)	0 (0%)	0.303
Diabetic ketoacidosis	0 (0%)	0 (0%)	-
Hypotension	1 (5.88%)	0 (0%)	0.303
Amputation	0 (0%)	0 (0%)	-
Genital infection	0 (0%)	0 (0%)	-

**Table 2:** Effect of dapagliflozin on fibrosis biomarkers PICP, MMP-1/TIMP-1 ratio and TGF-β1.

	PICP (ng/mL)			MMP-1/TIMP-1 Ratio			TGF-β1 (pg/mL)		
	Dapagliflozin Group	Control Group	*p*-Value	Dapagliflozin Group	Control Group	*p*-Value	Dapagliflozin Group	Control Group	*p*-Value
Pre-intervention	67.23 ± 44.61	56.04 ± 22.66	0.614	0.63 ± 0.31	0.73 ± 0.75	0.540	1.66 ± 0.64	1.39 ± 0.49	0.195
Post-intervention	158.54 ± 71.18	161.45 ± 107.63	0.719	0.32 ± 0.23	0.23 ± 0.16	0.171	3.38 ± 2.26	2.25 ± 1.77	0.058
*p*-value (post-pre)	0.000	0.000		0.005	0.002		0.005	0.044	
Delta (Δ)	91.30 ± 59.83	105.41 ± 94.88	0.943	0.31 ± 0.35	0.50 ± 0.68	0.885	1.73 ± 2.34	0.86 ± 1.77	0.207

**Table 3:** Effect of dapagliflozin on NT-pro BNP levels.

	Mitral-Valve Mean Pressure Gradient (pg/mL)		*p*-Value
	Dapagliflozin Group	Control Group	
Pre-intervention	7045.29 ± 3182.26	6928.12 ± 3690.44	0.857
Post-intervention	3210.88 ± 1019.46	4971.87 ± 3634.65	0.449
*p*-value (post-pre)	0.000	0.002	
Delta (Δ)	3832.42 ± 2857.52	1956.25 ± 1755.42	0.034

**Table 4:** Echocardiography parameters pre- and post-intervention.

Echocardiography Parameters	Dapagliflozin Group (*n* = 17)			Control Group (*n* = 16)			*p*-Value (between Pre-Intervention Group)	*p*-Value (between Post-Intervention Group)
	Pre-Intervention	Post-Intervention	*p*	Pre-Intervention	Post-Intervention	*p*		
MVA planimetry, cm^2^	0.75 ± 0.13	0.73 ± 0.03	0.403	0.77 ± 0.13	0.72 ± 0.03	0.099	0.616	0.905
LA diameter, mm	56.14 ± 9.26	56.06 ± 1.84	0.529	53.52 ± 8.36	53.72 ± 2.11	0.702	0.402	0.952
RV diameter, mm	36.29 ± 4.51	34.58 ± 4.95	0.057	33.46 ± 7.09	34.09 ± 1.14	0.679	0.177	0.822
LVIDd, mm	47.34 ± 7.21	49.34 ± 6.21	0.091	45.99 ± 5.81	45.14 ± 5.91	0.275	0.665	0.113
LAVI, mL/m^2^	145.89 ± 75.29	139.26 ± 69.27	0.055	139.26 ± 69.27	149.63 ± 105.89	0.326	0.707	0.744
Cn, mL/mmHg	4.82 ± 1.71	5.73 ± 2.19	0.017	5.21 ± 1.99	4.68 ± 1.73	0.379	0.546	0.121
Mean pressure gradient mitral, mmHg	13.23 ± 4.50	9.88 ± 3.87	0.001	12.35 ± 4.48	11.88 ± 4.01	0.756	0.579	0.155
Systolic pulmonary artery pressure, mmHg	64.83 ± 14.74	67.89 ± 9.78	0.446	65.65 ± 22.79	73.49 ± 22.32	0.335	0.902	0.353
LVEF, %	53.73 ± 10.36	50.31 ± 8.75	0.215	57.20 ± 9.02	56.02 ± 6.96	0.682	0.314	0.046
TAPSE, mm	18.30 ± 4.12	17.51 ± 3.76	0.568	18.42 ± 7.53	18.00 ± 4.22	0.480	0.954	0.423
Pulmonary hypertension probability (intermediate to high), %	15 (88.23%)	14 (82.35%)	1.00	12 (75.00%)	11 (64.71%)	1.00	0.398	0.362

**Table 5:** Effect of dapagliflozin on Cn value.

	Cn (mL/mmHg)		*p*-Value
	Dapagliflozin Group	Control Group	
Pre-intervention	4.82 ± 1.71	5.21 ± 1.99	0.546
Post-intervention	5.73 ± 2.19	4.68 ± 1.73	0.121
*p*-value (post-pre)	0.017	0.379	
Delta (Δ)	0.90 ± 1.29	−0.53 ± 2.57	0.049

**Table 6:** Effect of dapagliflozin on MPG mitral valve.

	Mitral Valve Mean Pressure Gradient (mmHg)		*p*-Value
	Dapagliflozin Group	Control Group	
Pre-intervention	13.23 ± 4.50	12.35 ± 4.48	0.579
Post-intervention	9.88 ± 3.87	11.88 ± 4.01	0.155
*p*-value (post-pre)	0.001	0.756	
Delta (Δ)	3.34 ± 3.11	0.46 ± 4.15	0.031

## Data Availability

All data are contained within the article.
